# P166: Improving children's and their visitors' hand hygiene compliance

**DOI:** 10.1186/2047-2994-2-S1-P166

**Published:** 2013-06-20

**Authors:** D Lary, K Hardie, J Randle

**Affiliations:** 1 School of Molecular Medical Sciences, University of Nottingham, Nottingham, UK; 2 School of Nursing, Midwifery and Physiotherapy, University of Nottingham, Nottingham, UK

## Introduction

Numerous interventions have aimed to improve the hand hygiene practices of healthcare workers in healthcare settings, however little attention has been paid to patients’ and their visitors’ hand hygiene. Children specifically are vulnerable to healthcare-associated infections, and again few studies have reported on their hand hygiene compliance.

## Objectives

The aim of the study was to increase children’s and their visitors’ hand hygiene compliance by an interactive educational intervention.

## Methods

The study was a cluster randomized control and multi-methods intervention trial involving; baseline and post-intervention hand hygiene observations, interactive educational activities using a novel hand hygiene training aids ‘Glo-Yo’ and mobile learning technology, and questionnaires and interviews.

## Results

Hand hygiene compliance following the intervention increased by 8.5% (P <0.001) compared with hand hygiene compliance before the intervention. There was no difference in compliance between children and their visitors (22% vs 28%, P= 0.051). While hand hygiene compliance varied depending on which of the five moments of hygiene undertaken (P<0.001), with highest compliance after body fluid exposure 65% (11/17); before patient contact 31% (86/93); after patient contact 22% (50/225) and after contact with surroundings 24% (13/54). Regarding the intervention sessions, 67% of the Glo-Yo group and 55% of mobile learning technology group has strongly agreed that the session was successful at raising awareness of the importance of hand hygiene compared to 30% in the control group. Additionally, 86% of visitors strongly agreed that the Glo-yo session has increased their child’s knowledge/understanding of when to wash hands and parts of hands that are difficult to wash compared to MLT and control group.

## Conclusion

There was evidence of a significant increase of HH compliance of patients and visitors during and post intervention (P <0.001) and that the Glo-yo session was successful at raising awareness of the importance of HH compared to the MLT and control group.

## Disclosure of interest

None declared

